# Arginine and homoarginine as predictors of cardiovascular mortality in chronic coronary syndrome

**DOI:** 10.1016/j.athplu.2026.100621

**Published:** 2026-10-23

**Authors:** Silje Kjellevold Storesund, Iman Karaji, Adrian McCann, Gard Frodahl Tveitevåg Svingen, Per Magne Ueland, Mai Tone Lønnebakken, Ottar Nygård, Eva Ringdal Pedersen

**Affiliations:** a Department of Heart Disease, Haukeland University Hospital, Bergen, Norway; b Department of Clinical Science, University of Bergen, Bergen, Norway; c Bevital A/S, Bergen, Norway

## Abstract

**Background and aims:**

Arginine (Arg) and homoarginine (hArg) are related to the nitric oxide pathway, which is important for vascular function. We investigated if serum concentrations of Arg and hArg predicted long-term cardiovascular disease (CVD) mortality in patients with chronic coronary syndrome (CCS), and whether associations differed between non-obstructive coronary artery disease (NOCAD) and obstructive coronary artery disease (OCAD).

**Methods and results:**

We included 4164 patients undergoing invasive coronary angiography for suspected CCS. Associations with CVD mortality were assessed using multivariable Cox regression, reporting hazard ratios (HRs) per standard deviation increase in log-transformed concentrations. During a median follow-up of 14.1 years, 586 (14.1%) patients died from CVD. Serum hArg was inversely associated with CVD mortality [HR (95% CI): 0.81 (0.74–0.88)] as was serum Arg [HR (95% CI): 0.90 (0.83–0.98)]. However, in Fine–Gray competing risk analyses, the association of serum Arg with CVD mortality was no longer statistically significant, whereas the inverse association for serum hArg persisted. Addition of serum hArg to the clinical model increased the C-statistic from 0.771 to 0.776, indicating a modest improvement in discrimination. In exploratory subgroup analyses, the association between serum hArg and CVD mortality was stronger in NOCAD than OCAD [0.54 (0.40–0.73) vs. 0.84 (0.77–0.92); P for interaction = 0.037].

**Conclusion:**

Serum Arg and hArg were inversely associated with long-term CVD mortality in patients with CCS. However, the association of serum hArg was more robust than that of serum Arg. The stronger association observed in NOCAD requires independent validation.

## Highlights

• Serum arginine and homoarginine associate with CVD risk in chronic coronary syndrome.
• CVD risk-association for homoarginine is stronger in non-obstructive coronary disease.
• Homoarginine adds incremental prognostic information beyond arginine.

## Graphical abstract

**Figure:**
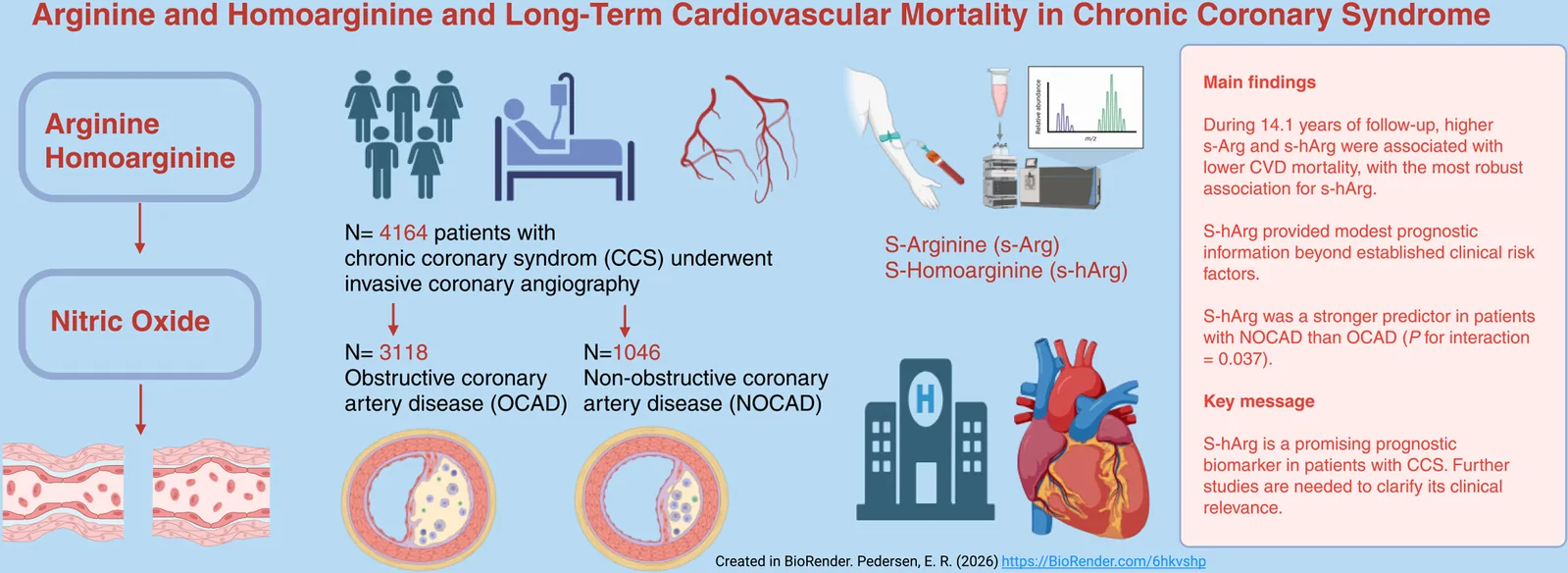

## 1 Introduction

Patients with chronic coronary syndrome (CCS) are frequently referred for invasive coronary angiography (ICA) because of chest pain and suspected myocardial ischemia, yet a substantial proportion do not have obstructive coronary artery disease (OCAD) [1], [2]. Non-obstructive coronary artery disease (NOCAD) is clinically important but heterogeneous ranging from angiographically normal coronary arteries to non-obstructive atherosclerotic disease [3]. Some patients may also have coronary microvascular or vasomotor dysfunction [3], which cannot by identified by conventional angiography alone. Despite this heterogeneity, patients with NOCAD overall have impaired cardiovascular prognosis, while tools for risk stratification remain limited [2], [3].

Nitric oxide (NO) is a central regulator of vascular tone and endothelial function [4], and impaired NO bioavailability is a hallmark of vascular dysfunction [4]. The amino acid arginine (Arg) and its closely related homologue, homoarginine (hArg), are integral components of the arginine–NO pathway and serve as substrates or modulators of NO synthesis [5], [6], [7], [8]. In addition, hArg has been suggested to influence endothelial function through alternative pathways [7], [9], including modulation of key enzymes such as arginase [10].

Circulating concentrations of Arg and hArg have been associated with adverse cardiovascular outcomes and mortality in both clinical and epidemiological studies [11], [12], [13], [14], [15], supporting their potential as biomarkers of vascular dysfunction. However, their prognostic relevance in patients with CCS remains largely unexplored, particularly in the context of different coronary artery disease (CAD) phenotypes.

Therefore, we measured serum concentrations of Arg and hArg in 4164 patients undergoing ICA for CCS, including both patients with NOCAD and OCAD, and evaluated their associations with long-term cardiovascular disease (CVD)- and all-cause mortality. In post hoc analyses, we also assessed associations of serum Arg and hArg with acute myocardial infarction (AMI).

## 2 Materials and methods

### 2.1 Study population and baseline characteristics

The study population and baseline characteristics have been described in more detail previously [16], [17], [18]. This prospective cohort study is based on the Western Norway Coronary Angiography Cohort (WECAC), which included 4164 patients undergoing ICA at Haukeland and Stavanger University hospitals in Norway due to CCS in the period between 2000 until 2004. Based on ICA, a total of 1046 patients were found to have NOCAD, defined by either the presence of only epicardial stenoses with <50% luminal narrowing, or absence of angiographically verified atherosclerotic plaques. The remaining participants (n = 3118) had OCAD, defined by at least one epicardial stenosis with ≥ 50% luminal narrowing. Patient-administered questionnaires were used to collect supplementary information on traditional risk factors, medications, and medical history as previously described [19]. Venous blood samples were collected and processed by trained personnel, then immediately frozen and biobanked at −80 °C until analyses in 2009. Serum Arg and hArg were measured by liquid chromatography-tandem mass spectrometry at Bevital AS, Bergen. The limit of detection for serum Arg and hArg were 0.25 and 0.10 μmol/L, respectively, while within- and between-day coefficient of variation (CV) was 4.5% and 5.5% for serum Arg and 5.9% and 8% for serum hArg [20].

Levels of c-reactive protein (CRP), LDL-cholesterol, HbA1c, hemoglobin, mean corpuscular volume, mean corpuscular hemoglobin concentration, fibrinogen, urate, leukocyte count, platelet count and creatinine were measured in fresh samples according to standard routines at the recruiting hospitals. Written Informed consents were provided by all participants. The study was approved by the Norwegian Data Protection Authority and the Regional Committee for Medical and Health Research Ethics (REK 2012/2167 and REK 2013/2324) and it was conducted in accordance with the declaration of Helsinki.

### 2.2 Follow-up and clinical end points

The study participants were followed from baseline until death or incident AMI, with censoring on 31 December 2017. We primarily assessed the associations with CVD- and all-cause mortality. Associations with incident AMI were explored in secondary, hypothesis-generating analyses to evaluate whether the findings were driven by atherothrombotic disease or reflected other aspects of vascular health.

CVD mortality was defined as causes of death coded I00–I99 according to the *International Statistical Classification of Diseases and Related Health Problems,* 10th *Revision* (ICD-10). AMI included fatal or non-fatal events coded I21 or I22 in hospital discharge summaries or death certificates.

Information on clinical events during follow-up was obtained through linkage to the Norwegian Cause of Death Registry and the Norwegian Patient Registry at the Norwegian Institute of Public Health.

### 2.3 Statistical analysis

Continuous variables are presented as medians (25th-75 ^th^ percentiles) and categorical variables as counts (percentages). Group comparisons were performed using the Mann–Whitney *U* test, or χ^2^ test, as appropriate. Correlations of serum Arg and hArg with clinical and blood characteristics were assessed using Spearman's rank correlation.

Associations of serum Arg and hArg with clinical outcomes were assessed using Cox proportional hazards regression. The multivariable models were adjusted for age, sex, BMI, HbA1c, LDL, smoking, hypertension, previous acute myocardial infarction, and estimated GFR. Additional sensitivity analyses included further adjustment for CRP, statin, beta blocker, aspirin and previous stroke. Baseline missingness was low: 4106 of 4164 participants (98.6%) had complete data for the multivariable serum Arg analyses and 4091 (98.2%) for the corresponding serum hArg analyses. Missing data were therefore handled by listwise deletion, and no imputation was performed. Effect modification by CAD status (NOCAD vs. OCAD) was assessed by including interaction terms.

The proportional hazards assumption was assessed using Schoenfeld residuals. Where evidence of non-proportionality was observed, sensitivity analyses were performed allowing time-varying effects for the affected covariate. Calibration of the fully adjusted Cox models was assessed at 10 years. Predicted 10-year event probabilities were obtained from the fitted Cox models, and observed 10-year event probabilities were estimated using the Kaplan–Meier method across the range of predicted risks. For CVD mortality and AMI, competing events were treated as censoring, consistent with the cause-specific Cox models used for the primary analyses. Calibration was assessed separately for models including serum Arg and hArg. In addition, Fine–Gray competing-risk regression was performed as a sensitivity analysis, treating non-CVD death as the competing event for CVD mortality and death before AMI as the competing event for AMI. Results from the Fine–Gray models are reported as subdistribution hazard ratios (sHRs) with 95% confidence intervals.

Discrimination was assessed using C-statistic, model fit by AIC and likelihood-ratio test. Risk reclassification was evaluated by calculating continuous net reclassification improvement (NRI), with 95% CIs obtained by bootstrap resampling.

A two-sided P-value <0.05 was considered statistically significant throughout, with exception of the correlation analyses which, due to multiple testing, were considered significant when <0.001.

We used IBM SPSS Statistics Version 29 (Armonk, NY: IBM Corp) or *R version 4.5.2 for Windows (R: A Language and Environment for Statistical Computing, R Core Team, R Foundation for Statistical Computing, Vienna, Austria, 2025,* https://www.R-project.org*)* for all the statistical analyses.

## 3 Results

### 3.1 Characteristics of the study population at baseline

Median age of the total study population (25th-75 ^th^ percentiles) was 62 (55-70) years. A total of 2997 (71.9%) of the participants were men. 1046 (25.1%) patients had NOCAD, while 3118 (74.9%) had OCAD. Overall, the median serum concentrations of Arg and hArg were 78.1 and 1.94 μmol/L, respectively. Levels of both amino acids were significantly lower in patients with NOCAD versus OCAD. Further information on baseline characteristics stratified by OCAD/NOCAD status is reported in Table 1. Baseline characteristics according to mortality status are presented in the Supplement (Supplementary Table S1).

**Table 1:** Characteristics of the study population.

	***Total n = 4164***	***Obstructive coronary artery disease (n = 3118)***	***Non-obstructive coronary artery disease (n = 1046)***	***P***
***Demographics and risk factors***				
*Age*	62 (55-70)	63 (56-71)	58 (51-65)	<0.001
*Male sex (%)*	2997 (71.9)	2458 (78.8)	539 (51.5)	<0.001
*BMI (kg/m2)*	26 (24-28)	26 (24-28)	26 (23-28)	0.10
*Smoking (%)*	1078 (25.9)	808 (25.9)	270 (25.8)	0.96
*Hypertension (%)*	1948 (46.8)	1520 (48.7)	428 (40.9)	<0.001
*Systolic BP (mmHg)*	140 (126-154)	140 (127-155)	139 (125-150)	0.005
*Diastolic BP (mmHg)*	80 (75-88)	80 (75-88)	80 (75-88)	0.94
*Self-reported DM (%)*	496 (11.9)	421 (13.5)	75 (7.2)	<0.001
*Previous acute myocardial infarction (%)*	1680 (40.3)	1504 (48.2)	176 (16.8)	<0.001
***Lab***				
*Hemoglobin (g/dl)*	14.3 (13.4-15.1)	14.4 (13.5-15.1)	14.1 (13.3-15.0)	<0.001
*MCH (pg)*	30 (30-31)	30 (30-31)	30 (29-31)	0.30
*MCHC (g/dl)*	33.2 (32.6-33.8)	33.2 (32.6-33.9)	33.2 (32.6-33.8)	0.36
*MCV (fL)*	92 (89-94)	92 (89-94)	91 (89-94)	0.80
*P-Fibrinogen (g/L)*	3.6 (3.2-4.1)	3.6 (3.2-4.1)	3.5 (3.1-4.0)	<0.001
*B-Leukocytes (x 10*^*9*^*/L)*	6.8 (5.7-8.2)	6.9 (5.8-8.3)	6.5 (5.4-7.8)	<0.001
*B-Thrombocytes (x 10*^*9*^*/L)*	240 (205-281)	236 (201-277)	252 (216-296)	<0.001
*S-CRP (mg/L)*	1.78 (0.87-3.67)	1.81 (0.90-3.70)	1.67 (0.78-3.48)	0.033
*S-Urat (μmol/L))*	352 (299-408)	361 (307-415)	328 (279-387)	<0.001
*HbA1c (%)*	6.1 (5.4-6.9)	6.1 (5.3-6.9)	6.2 (5.5-6.9)	0.002
*S-Glucose (mmol/L)*	5.6 (5.1-6.6)	5.7 (5.1-6.8)	5.5 (4.9-6.3)	<0.001
*S-LDL-cholesterol (mmol/L)*	2.9 (2.4-3.7)	2.9 (2.3-3.6)	3.1 (2.5-3.9)	<0.001
*Estimated GFR (ml/min/1.73 m2)*	91 (78-99)	90 (76-98)	93 (81-102)	<0.001
*S-Creatinine μmol/L*	89 (81-98)	91 (82-100)	84 (77-93)	<0.001
*S-Arginine (μmol/L)*	78.1 (63.4-92.7)	78.7 (64.4-93.2)	76.6 (59.6-91.6)	<0.001
*S-Homoarginine (μmol/L)*	1.94 (1.52-2.46)	1.96 (1.54-2.50)	1.87 (1.47-2.38)	<0.001
***Medications***				
*Aspirin (%)*	3401 (81.6)	2823 (90.5)	578 (55.3)	<0.001
*Statins (%)*	3342 (80.2)	2784 (89.2)	558 (53.3)	<0.001
*Beta blockers (%)*	3017 (72.4)	2494 (79.9)	523 (50.0)	<0.001
***Clinical endpoints during follow-up***				
*CVD mortality (%)*	586 (14.1)	526 (16.9)	60 (5.7)	<0.001
*All-cause mortality (%)*	1330 (31.9)	1149(36.8)	181(17.3)	<0.001
*Acute myocardial infarction (%)*	675 (16.2)	606 (19.4)	69 (6.6)	<0.001

Continuous variables are presented as median (25th-75 ^th^ percentile). Categorical variables are presented as numbers (%).

BMI, body mass index; BP, blood pressure; DM, diabetes mellitus; MCH, Mean corpuscular hemoglobin; MCHC, Mean corpuscular hemoglobin concentration; MCV, Mean corpuscular volume; CRP, C-reactive protein; LDL, low-density lipoprotein; GFR, glomerular filtration rate; CVD, cardiovascular disease.

### 3.2 Correlation analysis

Serum Arg and hArg were moderately positively inter-correlated (r: 0.23). Both serum Arg and hArg showed weak inverse correlations with plasma fibrinogen and CRP (r: −0.07 to −0.14). In addition, serum Arg was weakly inversely correlated with diastolic blood pressure and body mass index (BMI) (r: −0.05 to −0.07).

In contrast, serum hArg was weakly positively correlated with diastolic blood pressure and BMI (r: 0.06 to 0.23) and showed additional positive correlations with glucose (r: 0.11) and urate (r: 0.14). Interestingly, serum hArg was moderately positively correlated with hemoglobin (r: 0.29) and mean corpuscular hemoglobin concentration (r: 0.17).

Conversely, weak negative correlations (r: −0.07 to −0.11) were observed between serum hArg and several other hematological variables, including leukocyte count, mean corpuscular volume, and platelet count (all P < 0.001).

### 3.3 Associations of serum Arg and hArg with long-term clinical outcomes

During a median follow-up of 14.1 (25th-75 ^th^ percentiles: 13.2–15.4) years, a total of 1330 (31.9%) participants died, of whom 586 (14.1%) died from CVD. A total of 675 (16.2%) experienced a fatal or non-fatal AMI. As expected, clinical events occurred more frequently in patients with OCAD than in those with NOCAD.

In age- and sex-adjusted Cox regression analyses, higher serum concentrations of both Arg and hArg were associated with lower risk of CVD mortality, with hazard ratios (HRs) (95% confidence interval [CI]) per standard deviation increase of 0.91 (0.84–0.99; P = 0.030) and 0.83 (0.77–0.89; P < 0.001), respectively. These associations remained significant after multivariable adjustment (Table 2), with HRs (95% CI) of 0.90 (0.83–0.98; P = 0.010) for serum Arg and 0.81 (0.74–0.88; P < 0.001) for serum hArg.

**Table 2:** Multivariable adjusted^a^ HRs (95% CIs) for CVD mortality and all-cause mortality per 1 SD increment of (log transformed) s-arginine and s-homoarginine (n = 4164).

	*CVD mortality*		*All-cause mortality*	
	*HR (95% CI)*	*P*	*HR (95% CI)*	*P*
***S-Arginine***	0.90 (0.83-0.98)	0.010	0.91 (0.86-0.96)	<0.001
***S-Homoarginine***	0.81 (0.74-0.88)	<0.001	0.82 (0.77-0.86)	<0.001

a Adjusted for age, sex, BMI, HbA1c, LDL, smoking, hypertension, previous acute myocardial infarction, estimated GFR.

Fig. 1 visualizes the associations of serum Arg and hArg to CVD mortality stratified for CAD phenotypes. Serum hArg was a significantly stronger predictor of CVD mortality in patients with NOCAD compared with those with OCAD (P for interaction = 0.037; Table 3). A similar pattern was observed for serum Arg, although the interaction did not reach statistical significance (P for interaction = 0.09). Serum Arg and hArg were also inversely associated with all-cause mortality (Table 2), with multivariable-adjusted HRs (95% CI) of 0.91 (0.86–0.96) and 0.82 (0.77–0.86), respectively, but without significant effect modification by CAD status (P for interaction ≥ 0.74, Table 3). The interaction analyses were exploratory and were not adjusted for multiple testing.

**Fig. 1:**
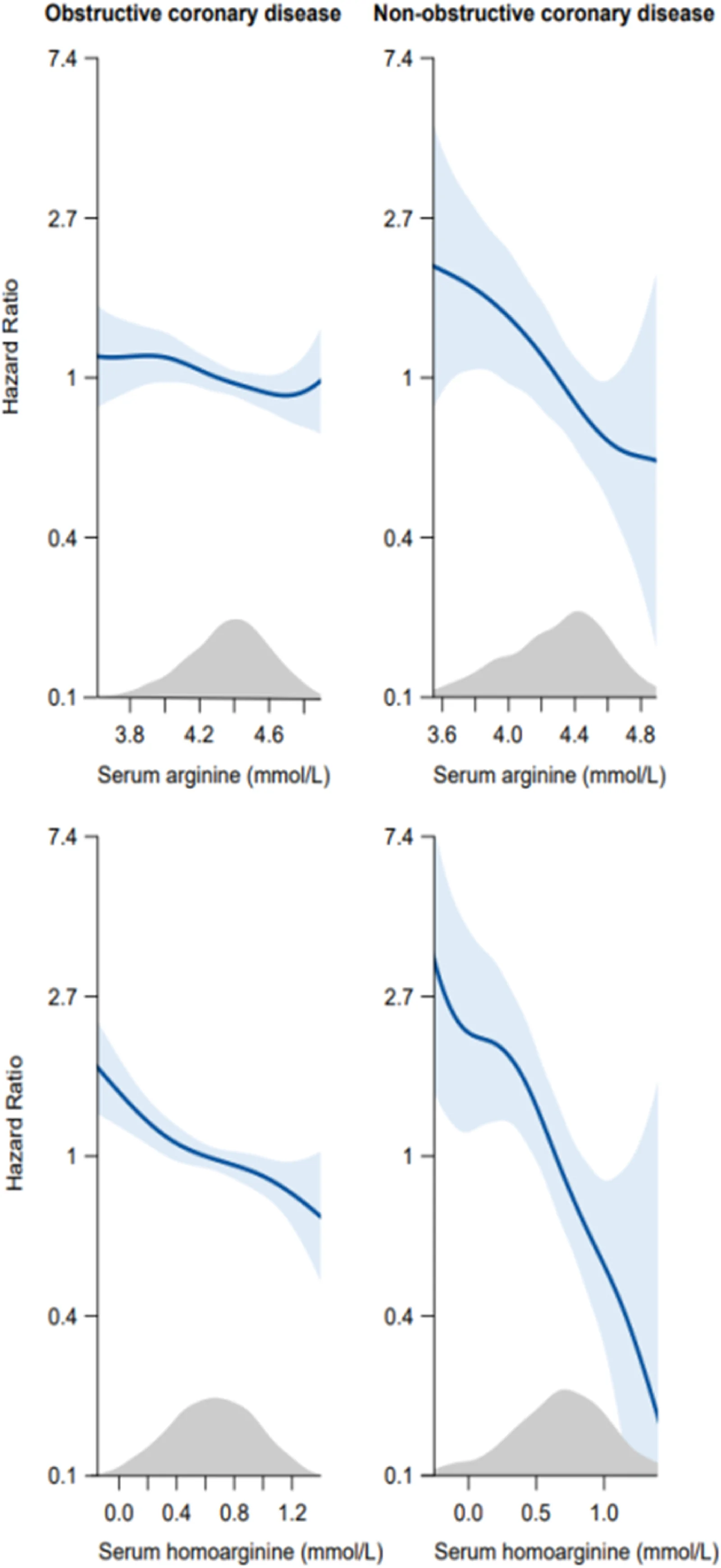
Dose-response relationship between serum Arg and hArg and risk of CVD mortality in OCAD versus NOCAD. Generalized additive regression models visualizing the dose-response relationship of serum Arg and hArg with CVD mortality in OCAD versus NOCAD. The models are adjusted for age, sex, BMI, HbA1c, LDL, smoking, hypertension, previous acute myocardial infarction, estimated GFR. The HR and the 95% CIs are visualized through the solid lines and the shaded areas, respectively, and the distribution of the metabolites are illustrated in the density plots.

**Table 3:** Multivariable adjusted^a^ HRs (95% CIs) for CVD mortality and all-cause mortality per 1 SD increment of (log transformed) s-arginine and s-homoarginine in OCAD (n = 3118) versus NOCAD (n = 1046).

	*OCAD*	*NOCAD*	*P int*
	*HR (95% CI)*	*HR (95% CI)*	
***S-Arginine***			
*CVD mortality*	0.93 (0.85-1.01)	0.71 (0.56-0.90)	0.09
*All-cause mortality*	0.91 (0.86-0.96)	0.90 (0.78-1.03)	0.74
***S-Homoarginine***			
*CVD mortality*	0.84 (0.77-0.92)	0.54 (0.40-0.73)	0.037
*All-cause mortality*	0.82 (0.77-0.87)	0.80 (0.67-0.94)	0.80

a Adjusted for age, sex, BMI, HbA1c, LDL, smoking, hypertension, previous acute myocardial infarction, estimated GFR.

Both serum Arg and hArg were modestly associated with lower risk of incident AMI (Supplementary Table S2), with multivariable-adjusted HRs (95% CI) of 0.92 (0.85–0.99) and 0.88 (0.81–0.95), respectively (P ≤ 0.03). For AMI there was no significant interaction according to CAD status (P for interaction ≥ 0.67; Supplementary Table S3).

Results were also similar in an extended multivariable model including additional adjustment for baseline medications, CRP and previous stroke (Supplementary Tables S4–S7).

When both biomarkers were included in the same multivariate Cox model, serum hArg remained associated with CVD mortality [HR 0.82 (95% CI 0.76–0.90); P < 0.001], whereas serum Arg did not [HR 0.94 (95% CI 0.86–1.02); P = 0.13].

The proportional hazards assumption was satisfied for serum hArg in relation to CVD mortality and for both serum Arg and hArg in relation to incident AMI. For serum Arg and CVD mortality, evidence of non-proportionality was observed, with the inverse association being stronger early during follow-up and attenuating over time (P = 0.007 for the time-dependent effect). However, the multivariable risk estimate of serum Arg remained basically unchanged in sensitivity analyses allowing time-varying effects [HR 0.92 (95% CI 0.85–0.99); P = 0.037].

Fine–Gray competing-risk analyses generally supported the main findings for serum hArg. Higher serum hArg was associated with lower risk of both CVD mortality [sHR 0.89 (95% CI 0.82–0.96); P = 0.004] and AMI [sHR 0.91 (95% CI 0.84–0.98); P = 0.017]. In contrast, the corresponding associations for serum Arg were attenuated and no longer statistically significant for either CVD mortality [sHR 0.93 (95% CI 0.85–1.01); P = 0.086] or AMI [sHR 0.94 (95% CI 0.88–1.02); P = 0.13] (Supplementary Table S8).

### 3.4 Calibration, model fit, discrimination and reclassification

Ten-year calibration plots showed generally reasonable agreement between predicted and observed risks, particular for all-cause mortality, although some overestimation was seen at higher predicted risk levels for CVD mortality and AMI (Supplementary Fig. S1).

Model fit improved with the addition of either serum Arg or hArg, whereas improvements in discrimination were modest (Supplementary Table S9). Adding serum hArg to the clinical model increased the C-statistic for CVD mortality from 0.771 to 0.776, P = 0.020. No significant improvement in C-statistic was found for serum Arg. As changes in C-statistic may be small despite incremental prognostic information, we also assessed risk classification using continuous NRI after adding serum Arg or hArg to the clinical model within CAD phenotypes. The results are summarized in Fig. 2.

**Fig. 2:**
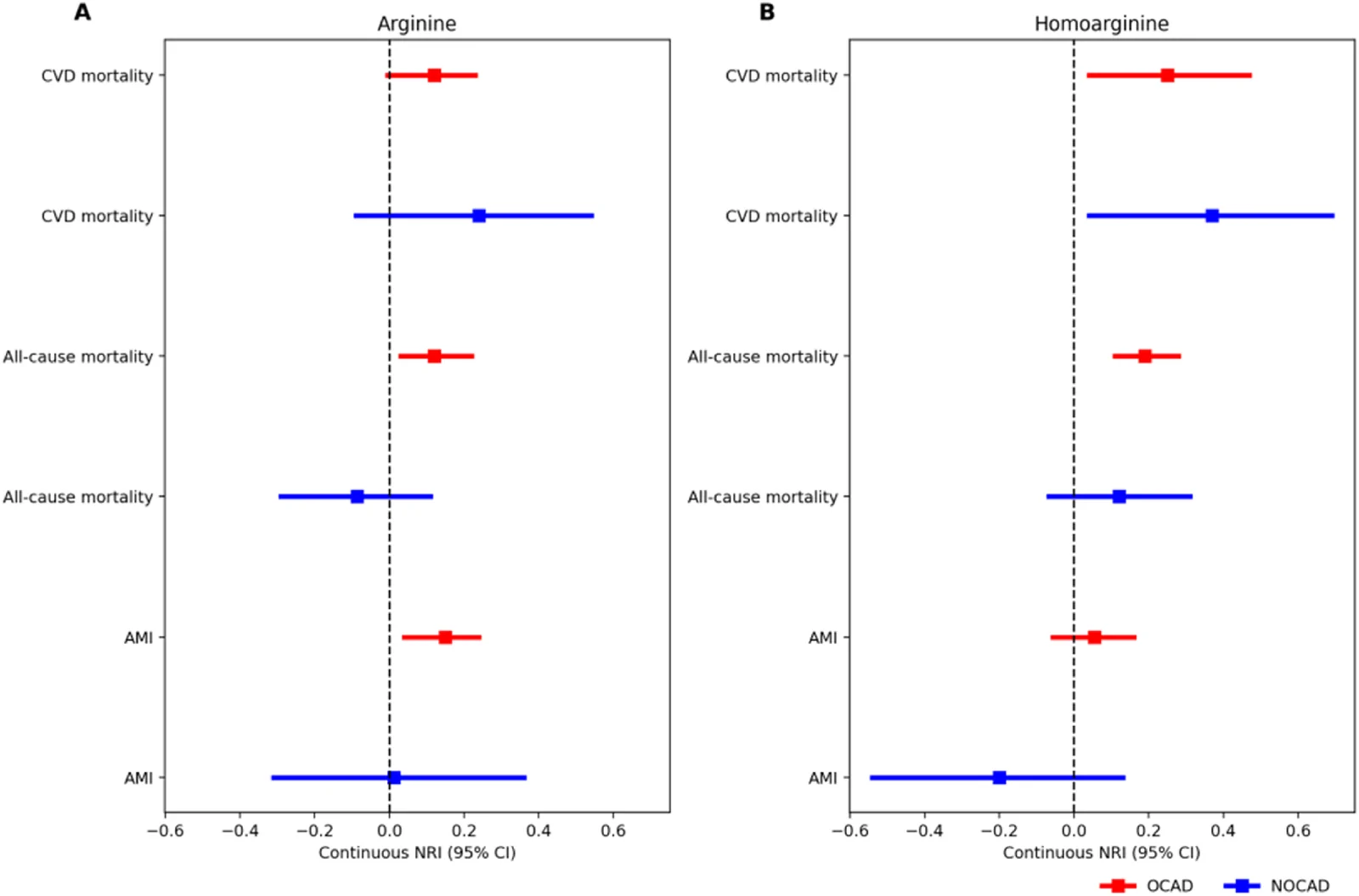
Continuous net reclassification improvement according to coronary phenotype and clinical endpoint. Continuous net reclassification improvement (NRI) with 95% bootstrap confidence intervals by adding serum arginine (left panel) and serum homoarginine (right panel) to a multivariable Cox regression model including the following covariables: age, sex, BMI, HbA1c, LDL, smoking, hypertension, previous acute myocardial infarction, estimated GFR. Analyses were stratified by coronary phenotype (obstructive coronary artery disease [OCAD] and non-obstructive coronary artery disease [NOCAD]) and evaluated for three outcomes: all-cause mortality, cardiovascular mortality, and acute myocardial infarction. Points represent NRI estimates and horizontal lines denote 95% confidence intervals derived from 1000 bootstrap iterations. The vertical dashed line indicates no improvement in reclassification (NRI = 0). Positive values reflect improved risk reclassification with inclusion of the biomarker.

For CVD mortality, serum hArg improved reclassification in both OCAD [NRI 0.25 (0.04–0.47), P = 0.009] and NOCAD [NRI 0.37 (0.04–0.69), P = 0.027] while serum Arg did not significantly improve reclassification in either group (Supplementary Table S10).

For all-cause mortality (Supplementary Table S11), both serum Arg and hArg improved reclassification in patients with OCAD [serum Arg: NRI 0.12 (95% CI 0.03–0.22), P = 0.004; serum hArg: NRI 0.19 (0.11–0.28), P < 0.001], whereas no significant improvement was observed in NOCAD.

For incident AMI, serum Arg improved reclassification in OCAD [NRI 0.15 (0.04–0.24), P = 0.01], whereas no significant improvement was observed in NOCAD. Serum hArg did not improve reclassification for AMI in either subgroup (Supplementary Table S12).

## 4 Discussion

### 4.1 Principal findings

In this large prospective cohort of patients with CCS, both serum Arg and hArg were inversely associated with CVD mortality and all-cause mortality, and more modestly with incident AMI. The association between serum hArg and CVD mortality was stronger in patients with NOCAD than in those with OCAD. When both biomarkers were included in the same model, serum hArg remained associated with CVD mortality, whereas the association with serum Arg was attenuated. Thus, serum hArg showed a more robust prognostic association than serum Arg, although the improvement in discrimination was small. The interaction according to CAD phenotype should be interpreted cautiously because it was modest, was not adjusted for multiple testing, and was confined to CVD mortality. Competing-risk analyses supported the main findings for serum hArg, whereas the associations for serum Arg were attenuated and no longer statistically significant. The observed overestimation at higher predicted risks for CVD mortality and AMI further indicates that the absolute risk estimates from these models should be interpreted cautiously.

### 4.2 A**rginine, homoarginine and epidemiological findings**

Accumulating evidence suggests that amino acid metabolism is closely linked to CVD [21]. Arg has been extensively studied in experimental studies due to its role as a precursor of NO [22], and a growing body of evidence supports a robust association between hArg and CVD risk. A cohort based on the Ludwigshafen Risk and Cardiovascular Health study, reported low serum hArg concentrations were associated with an increased risk of both CVD and all-cause mortality [7]. Similar findings have been reported in patients with renal disease [23], elderly populations [24], and community based cohorts [13].

An important aspect of the present study is the direct comparison of serum Arg and hArg within the same well-characterized cohort. Previous prospective studies have primarily focused on hArg and, to a lesser extent on Arg [25]. Our findings extend previous observations by showing that, when the two biomarkers were considered together, serum hArg retained an association with CVD mortality and added modest prognostic information beyond serum Arg. The stronger association of serum hArg with CVD mortality in NOCAD is also of interest. To the best of our knowledge, our study is the first to examine serum levels of Arg and hArg specifically in patients with NOCAD. Our findings nevertheless should be interpreted cautiously and requires independent validation.

### 4.3 Arginine, homoarginine, nitric oxide and vascular biology

The stronger risk estimates of serum Arg and hArg to CVD mortality than with incident AMI may suggest that these amino acids reflect broader aspects of vascular health rather than atherothrombotic disease alone.

Arg is a key substrate for nitric oxide synthase (NOS) and an important precursor of NO, a central regulator of vascular tone and endothelial function [4]. It is derived from endogenous synthesis, protein catabolism, and dietary intake, and is metabolized through several pathways, including the arginase and NOS pathways [5], [26].

The structurally related amino acid hArg is synthesized mainly from lysine and Arg. HArg may influence NO availability both as a weak substrate for NOS and through inhibition of arginase [8], [10], [27], thereby increasing arginine bioavailability. In addition, hArg has been linked to other vasoprotective mechanisms, including inhibition of tissue-nonspecific alkaline phosphatase, which is involved in vascular calcification [28]. Taken together, these mechanisms suggest that Arg and hArg, although closely related, may capture different aspects of vascular biology.

The observed correlations between serum hArg and inflammatory, hematological, and metabolic biomarkers support the hypothesis that hArg reflects a broader pathophysiological state related to endothelial function. Potentially, the stronger inverse associations of serum hArg to CVD mortality, particularly in NOCAD, may reflect additional vasoprotective mechanisms beyond NO availability [12], [28].

Circulating Arg levels are influenced by multiple metabolic pathways and may be more susceptible to dietary variation than hArg, which could theoretically limit its prognostic utility [24]. However, we have previously demonstrated moderate-to-good within-person reproducibility for both biomarkers, with similar intraclass correlation coefficients for serum Arg and hArg [29], [30]. Thus, differences in biomarker stability are unlikely to explain the observed differences in their associations with cardiovascular outcomes.

### 4.4 Clinical implications

Patients with NOCAD represent a large and heterogeneous group in whom risk stratification remains challenging. In the present study, both serum Arg and hArg were inversely associated with CVD mortality in this subgroup, with the strongest association observed for serum hArg.

Continuous NRI analyses suggested that adding serum hArg improved risk reclassification for CVD mortality; however, the absolute gains in discrimination were small, and improvements in risk classification were not consistent across endpoints. The present findings therefore support an independent prognostic association and provide a basis for further studies.

External validation, including contemporary cohorts with more detailed characterization of NOCAD, is needed before any potential clinical relevance of hArg can be established.

### 4.5 Strengths and limitations

This study has several strengths, including a large sample size, long-term follow-up, and detailed baseline characterization, and simultaneous targeted measurement of serum Arg and hArg. The direct comparison of the two biomarkers and the comprehensive analyses of discrimination, calibration, and competing risks strengthen the assessment of their prognostic relevance.

Several limitations should also be acknowledged. First, the observational design precludes conclusions about causality, and residual confounding cannot be excluded despite extensive adjustment and sensitivity analyses. Second, serum Arg and hArg were measured only once at baseline, and temporal changes could not be assessed, although previous data indicate comparable within-person reproducibility for the two biomarkers. Third, our NOCAD definition combined angiographically normal coronary arteries and non-obstructive atherosclerotic disease, and no assessment of coronary microvascular or vasomotor dysfunction was available. The NOCAD group should therefore not be interpreted as a homogeneous phenotype. In addition, subgroup analyses according to CAD phenotype were exploratory and were not adjusted for multiple comparisons. Fourth, causes of death were based on registry ICD-10 codes rather than adjudication, and some outcome misclassification is possible. Finally, the cohort was recruited in 2000 – 2004, before contemporary diagnostic approaches and preventive treatment, which may limit generalizability to current CCS populations.

### 4.6 Conclusion

In patients with CCS, higher circulating concentrations of both Arg and hArg were associated with lower long-term risk of CVD mortality. However, the association for serum hArg was most robust and provided incremental prognistic information beyond serum Arg. The risk-association of serum hArg also appeared stronger in NOCAD than in OCAD. Nevertheless, the improvement in discrimination was small. Further studies are needed to validate these findings and clarify their clinical relevance.

## Author contributions

S.K.S. and E.R.P. performed the statistical analysis and wrote the manuscript. P.M.U. and A.M. supervised the analysis of the included metabolites. E.R.P., G.F.T.S. and O.N. collected and processed clinical data. S.K.S., I.K., A.M., G.F.T.S., P.M.U., M.T.L., O.N., and E.R.P. revised the manuscript. S.K.S. is the guarantor of this work and, as such, had full access to all the data in the study and takes responsibility for the integrity of the data and the accuracy of the data analysis. All authors approved the final version of the manuscript.

## Ethics declaration

Written informed consent to take part in the study and to publish the article has been obtained from all participants or their legal representatives. The privacy rights of participants have been observed.

This study included organ or tissue donors. This study includes human biological material and consent was obtained by donors, or their next of kin or legal representatives, for use in this study and for publication of the article. The samples used in this research were not sourced from executed prisoners or prisoners of conscience.

This study was performed in compliance with relevant laws, regulatory frameworks and guidelines where the research took place. This study was approved by the Regional Committee for Medical and Health Research Ethics. (Approval No. REK 2012/2167 and REK 2013/2324)

## Funding

The funding of this study was ensured by the Norwegian 10.13039/501100003506Ministry of Health and Care Services, the Western Norway Regional Health Authority, the Department of Heart Disease, 10.13039/501100010586Haukeland University Hospital, Bergen, Norway, and the 10.13039/501100010698Norwegian Heart and Lung Patient Organisation. None of the funding institutions were involved in the study design, data collection, analysis and interpretation of data, writing, or in the decision to submit the paper.

## Declaration of competing interest

The authors declare the following financial interests/personal relationships which may be considered as potential competing interests: The authors have no competing interests and no editorial roles in relation to this journal.
