# Supplementary material for: Arginine and homoarginine as predictors of cardiovascular mortality in chronic coronary syndrome
Source: Atheroscler Plus. 2026 Oct 23;66:100621. doi: 10.1016/j.athplu.2026.100621 (PMC13721928; doi:10.1016/j.athplu.2026.100621)
Supplement: Supplementary file 1 [file mmc1.docx]

**Appendix A, Supplementary material**

| Supplementary Table S1. Baseline characteristics of the study population stratified by mortality during follow-up | | | | |
| --- | --- | --- | --- | --- |
|  | ***Total n=4164*** | ***Survivals***  ***n=2836*** | ***Diseased***  ***n=1330*** | ***P*** |
| ***Demographics and***  ***risk factors*** |  |  |  |  |
| *Age* | 62 (55-70) | 59 (53-66) | 70 (62-75) | <0.001 |
| *Male sex (%)* | 2997 (71.9) | 1980 (69.8) | 1017 (76.5) | <0.001 |
| *BMI (kg/m2)* | 26 (24-28) | 26 (24-28) | 26 (23-29) | 0.010 |
| *Smoking (%)* | 1078 (25.9) | 711 (25.1) | 367 (27.6) | 0.08 |
| *Hypertension (%)* | 1948 (46.8) | 1206 (42.5) | 742 (55.8) | <0.001 |
| *Systolic BP (mmHg)* | 140 (126-154) | 140 (125-153) | 140 (128-158) | <0.001 |
| *Diastolic BP (mmHg)* | 80 (75-88) | 80 (75-88) | 80 (74-89) | 0.12 |
| *Self-reported DM (%)* | 496 (11.9) | 260 (9.2) | 236 (17.7) | <0.001 |
| *Previous acute myocardial infarction (%)* | 1680 (40.3) | 1014 (35.8) | 666 (50.1) | <0.001 |
| ***Lab*** |  |  |  |  |
| *Hemoglobin (g/dl)* | 14.3 (13.4-15.1) | 14.4 (13.6-15.2) | 14.1 (13.1-15.0) | <0.001 |
| *MCH (pg)* | 30 (30-31) | 30 (30-31) | 30 (29-31) | 0.83 |
| *MCHC (g/dl)* | 33.2 (32.6-33.8) | 33.3 (32.7-33.9) | 33.0 (32.3-33.6) | <0.001 |
| *MCV (fL)* | 92 (89-94) | 91 (89-94) | 92 (89-95) | <0.001 |
| *P-Fibrinogen (g/L)* | 3.6 (3.2-4.1) | 3.5 (3.1-4.0) | 3.8 (3.3-4.3) | <0.001 |
| *B-Leukocytes*  *(x 10^9^/L)* | 6.8 (5.7-8.2) | 6.7 (5.6-8.0) | 7.2 (6.0-8.6) | <0.001 |
| *B-Thrombocytes*  *(x 10^9^/L)* | 240 (205-281) | 243 (207-283) | 233 (197-278) | <0.001 |
| *S-CRP (mg/L)* | 1.78 (0.87-3.67) | 1.60 (0.79-3.16) | 2.25 (1.12-4.97) | <0.001 |
| *S-Urat (µmol/L))* | 352 (299-408) | 346 (294-400) | 368 (312-429) | <0.001 |
| *HbA1c (%)* | 6.1 (5.4-6.9) | 6.1 (5.4-6.8) | 6.1 (5.4-7.0) | 0.012 |
| *S-Glucose (mmol/L)* | 5.6 (5.1-6.6) | 5.6 (5.1-6.4) | 5.8 (5.1-7.1) | <0.001 |
| *S-LDL*-*cholesterol (mmol/L)* | 2.9 (2.4-3.7) | 2.9 (2.4-3.7) | 2.9 (2.4-3.7) | 0.41 |
| *Estimated GFR (ml/min/1.73 m2)* | 91 (78-99) | 93 (84-101) | 83 (68-94) | <0.001 |
| *S-Creatinine µmol/L* | 89 (81-98) | 88 (80-96) | 93 (83-105) | <0.001 |
| *S-Arginine*  *(µmol/L)* | 78.1 (63.4-92.7) | 78.6 (64.1-92.9) | 77.0 (61.7-92.3) | 0.044 |
| *S-Homoarginine (µmol/L)* | 1.94 (1.52-2.46) | 1.99 (1.57-2.52) | 1.83 (1.42-2.30) | <0.001 |
| **Medications** |  |  |  |  |
| *Aspirin (%)* | 3401 (81.6) | 2301 (81.1) | 1100 (82.7) | 0.22 |
| *Statins (%)* | 3342 (80.2) | 2279 (80.4) | 1063 (79.9) | 0.74 |
|  |  |  |  |  |
| *Beta blockers (%)* | 3017 (72.4) | 2020 (71.2) | 997 (75) | 0.012 |
| **Clinical endpoints during follow-up** | | | | |
| *CVD mortality (%)* | 586 (14.1) | 60 (5.7) | 526 (16.9) | <0.001 |
| *All-cause mortality (%)* | 1330 (31.9) | 181 (17.3) | 1149 (36.8) | <0.001 |
| *Acute myocardial infarction (%)* | 675 (16.2) | 265 (9.3) | 410 (30.8) | <0.001 |
| Continuous variables are presented as median (25^th^-75 ^th^ percentile).  Categorical variables are presented as numbers (%).  BMI, body mass index; BP, blood pressure; DM, diabetes mellitus; MCH, Mean corpuscular hemoglobin; MCHC, Mean corpuscular hemoglobin concentration; MCV, Mean corpuscular volume; CRP, C-reactive protein; LDL, low-density lipoprotein; GFR, glomerular filtration rate; CVD,  cardiovascular disease. | | | | |

| Supplementary Table S2.  Multivariable adjusted* HRs (95 % CIs) for acute myocardial infarction per 1 SD increment of (log transformed) s-arginine and s-homoarginine (n=4164) | | | | | | |
| --- | --- | --- | --- | --- | --- | --- |
|  | ***HR (95 % CI)*** | | | ***P*** | | |
| ***S-Arginine*** | 0.92 (0.85-0.99) | | | 0.032 | | |
| ***S-Homoarginine*** | 0.88 (0.81-0.95) | | | 0.002 | | |
| *Adjusted for age, sex, BMI, HbA1c, LDL, smoking, hypertension, previous acute myocardial infarction, estimated GFR. | | | | | | |
| Supplementary Table S3.  Multivariable adjusted* HRs (95 % CIs) for acute myocardial infarction per 1 SD increment of (log transformed) s-arginine and s-homoarginine in OCAD (n=3118) versus NOCAD (n=1046) | | | | | |  |
|  | | ***OCAD*** | ***NOCAD*** | |  |  |
|  | | ***HR (95 % CI)*** | ***HR (95 % CI)*** | | ***P int*** |  |
| ***S-Arginine*** | |  |  | |  |  |
| *Acute myocardial infarction* | | 0.92 (0.84-0.99) | 0.90 (0.72-1.12) | | 0.83 |  |
|  | |  |  | |  |  |
| ***S-Homoarginine*** | |  |  | |  |  |
| *Acute myocardial infarction* | | 0.89 (0.81-0.97) | 0.84 (0.64-1.09) | | 0.67 |  |
| *Adjusted for age, sex, BMI, HbA1c, LDL, smoking, hypertension, previous acute myocardial infarction, estimated GFR. | | | | | |  |

| Supplementary Table S4.  Multivariable adjusted* HRs (95 % CIs) for CVD mortality and all-cause mortality per 1 SD increment of (log transformed) s-arginine and s-homoarginine (n=4164) | | | | |
| --- | --- | --- | --- | --- |
|  | ***CVD mortality*** |  | ***All-cause mortality*** | |
|  | ***HR (95 % CI)*** | ***P*** | ***HR (95 % CI)*** | ***P*** |
| ***S-Arginine*** | 0.92 (0.85-1.00) | 0.051 | 0.93 (0.88-0.980) | 0.007 |
| ***S-Homoarginine*** | 0.83 (0.76-0.91) | <0.001 | 0.83 (0.79-0.88) | <0.001 |
| *Adjusted for age, sex, BMI, HbA1c, LDL, smoking, hypertension, previous acute myocardial infarction, estimated GFR, CRP, statin, beta blocker, aspirin, previous stroke. | | | | |

| Supplementary Table S5.  Multivariable adjusted* HRs (95 % CIs) for CVD mortality and all-cause mortality per 1 SD increment of (log transformed) s-arginine and s-homoarginine in OCAD (n=3118) versus NOCAD (n=1046) | | | |
| --- | --- | --- | --- |
|  | ***OCAD*** | ***NOCAD*** |  |
|  | ***HR (95 % CI)*** | ***HR (95 % CI)*** | ***P int*** |
| ***S-Arginine*** |  |  |  |
| *CVD mortality* | 0.95 (0.87-1.04) | 0.75 (0.58-0.95) | 0.10 |
| *All-cause mortality* | 0.93 (0.87-0.98) | 0.93 (0.80-1.06) | 0.65 |
| ***S-Homoarginine*** |  |  |  |
| *CVD mortality* | 0.86 (0.79-0.95) | 0.58 (0.42-0.79) | 0.046 |
| *All-cause mortality* | 0.83 (0.79-0.89) | 0.85 (0.71-1.01) | 0.68 |
| *Adjusted for age, sex, BMI, HbA1c, LDL, smoking, hypertension, previous acute myocardial infarction, estimated GFR, CRP, statin, beta blocker, aspirin, previous stroke. | | | |

| Supplementary Table S6.  Multivariable adjusted* HRs (95 % CIs) for acute myocardial infarction per 1 SD increment of (log transformed) s-arginine and s-homoarginine (n=4164) | | |
| --- | --- | --- |
|  | ***HR (95 % CI)*** | ***P*** |
| ***S-Arginine*** | 0.93 (0.86-1.00) | 0.054 |
| ***S-Homoarginine*** | 0.89 (0.82-0.97) | 0.006 |
| *Adjusted for age, sex, BMI, HbA1c, LDL, smoking, hypertension, previous acute myocardial infarction, estimated GFR, CRP, statin, beta blocker, aspirin, previous stroke. | | |

| Supplementary Table S7.  Multivariable adjusted* HRs (95 % CIs) for acute myocardial infarction per 1 SD increment of (log transformed) s-arginine and s-homoarginine in OCAD (n=3118) versus NOCAD (n=1046) | | | |
| --- | --- | --- | --- |
|  | ***OCAD*** | ***NOCAD*** |  |
|  | ***HR (95 % CI)*** | ***HR (95 % CI)*** | ***P int*** |
| ***S-Arginine*** |  |  |  |
| *Acute myocardial infarction* | 0.93 (0.86-1.01) | 0.91 (0.73-1.14) | 0.77 |
| ***S-Homoarginine*** |  |  |  |
| *Acute myocardial infarction* | 0.90 (0.83-0.99) | 0.89 (0.68-1.18) | 0.73 |
| *Adjusted for age, sex, BMI, HbA1c, LDL, smoking, hypertension, previous acute myocardial infarction, estimated GFR, CRP, statin, beta blocker, aspirin, previous stroke. | | | |

Supplementary Table S8.

Multivariable adjusted* subdistribution HRs (95 % CIs) for CVD mortality and acute myocardial infarction per 1 SD increment of (log transformed) s-arginine and s-homoarginine (n=4106 for s-arginine and n=4091 for s-homoarginine)

|  | ***CVD mortality*** |  | ***Acute myocardial infarction*** | |
| --- | --- | --- | --- | --- |
|  | ***sHR (95 % CI)*** | ***P*** | ***sHR (95 % CI)*** | ***P*** |
| ***S-Arginine*** | 0.93 (0.85-1.01) | 0.086 | 0.94 (0.88-1.02) | 0.13 |
| ***S-Homoarginine*** | 0.89 (0.82-0.96) | 0.004 | 0.91 (0.84-0.98) | 0.017 |
| *Adjusted for age, sex, BMI, HbA1c, LDL, smoking, hypertension, previous acute myocardial infarction, and estimated GFR. Fine-Gray models accounted for non-CVD death as a competing event for CVD mortality and death before myocardial infarction as a competing event for acute myocardial infarction. | | | | |

| Supplementary Table S9.  Model performance for prediction of CVD mortality | | | | |
| --- | --- | --- | --- | --- |
| ***Model*** | ***C-statistic (SE)*** | ***P for change in discrimination**** | ***AIC*** | ***P for model fit†*** |
| ***Clinical model*** | 0.771 (0.010) | — | 8580.6 | — |
| ***Clinical + Arg*** | 0.773 (0.010) | 0.124 | 8576.0 | 0.010 |
| ***Clinical + hArg*** | 0.776 (0.010) | 0.020 | 8560.3 | <0.001 |
| ***Clinical + Arg + hArg*** | 0.776 (0.010) | 0.338 | 8560.0 | 0.13 |
| Higher C-statistic indicates better discrimination and lower AIC indicates better model fit.  *P values for change in discrimination are based on the corresponding comparison of 10-year AUC between models.  †P values for model fit are from likelihood-ratio tests. For models including s-Arg or s-hArg alone, the reference was the clinical model; for the model including both biomarkers, the reference was the clinical model + s-hArg. | | | | |

| Supplementary Table S10.  Net Reclassification Improvement – CVD mortality | | | | |
| --- | --- | --- | --- | --- |
|  | ***OCAD*** | | ***NOCAD*** | |
|  | ***NRI (95 % CI)*** | ***P*** | ***NRI (95 % CI)*** | ***P*** |
| ***S-Arginine*** | 0.12 (-0.005-0.23) | 0.058 | 0.24 (-0.09-0.54) | 0.14 |
| ***S-Homoarginine*** | 0.25 (0.04-0.47) | 0.009 | 0.37 (0.04-0.69) | 0.027 |
| *Adjusted for age, sex, BMI, HbA1c, LDL, smoking, hypertension, previous acute myocardial infarction, estimated GFR. | | | | |

| Supplementary Table S11.  Net Reclassification Improvement – All-cause mortality | | | | |
| --- | --- | --- | --- | --- |
|  | ***OCAD*** | | ***NOCAD*** | |
|  | ***NRI (95 % CI)*** | ***P*** | ***NRI (95 % CI)*** | ***P*** |
| ***S-Arginine*** | 0.12 (0.03-0.22) | 0.004 | -0.087 (-0.29-0.11) | 0.37 |
| ***S-Homoarginine*** | 0.19 (0.11-0.28) | <0.001 | 0.12 (-0.068-0.31) | 0.23 |
| *Adjusted for age, sex, BMI, HbA1c, LDL, smoking, hypertension, previous acute myocardial infarction, estimated GFR. | | | | |

| Supplementary Table S12.  Net Reclassification Improvement – Acute myocardial infarction | | | | |
| --- | --- | --- | --- | --- |
|  | ***OCAD*** | | ***NOCAD*** | |
|  | ***NRI (95 % CI)*** | ***P*** | ***NRI (95 % CI)*** | ***P*** |
| ***S-Arginine*** | 0.15 (0.04-0.24) | 0.01 | 0.011 (-0.31-0.36) | 0.90 |
| ***S-Homoarginine*** | 0.054 (-0.057-0.16) | 0.34 | -0.20 (-0.54-0.13) | 0.27 |
| *Adjusted for age, sex, BMI, HbA1c, LDL, smoking, hypertension, previous acute myocardial infarction, estimated GFR. | | | | |

Supplementary Figure S1.

Ten-year calibration of the fully adjusted Cox proportional hazards models.

Calibration plots compare predicted and observed 10-year risk for (A) s-arginine and all-cause mortality, (B) s-arginine and cardiovascular mortality, (C) s-arginine and acute myocardial infarction, (D) s-homoarginine and all-cause mortality, (E) s-homoarginine and cardiovascular mortality, and (F) s-homoarginine and acute myocardial infarction. Models were adjusted for age, sex, BMI, HbA1c, LDL cholesterol, smoking, hypertension, previous acute myocardial infarction, and estimated GFR. The dashed diagonal line represents perfect calibration. Axes are displayed from 0% to 80%; this range encompasses at least 99% of predicted 10-year risks in each model.
